# A novel species of the marine cyanobacterium *Acaryochloris* with a unique pigment content and lifestyle

**DOI:** 10.1038/s41598-018-27542-7

**Published:** 2018-06-14

**Authors:** Frédéric Partensky, Christophe Six, Morgane Ratin, Laurence Garczarek, Daniel Vaulot, Ian Probert, Alexandra Calteau, Priscillia Gourvil, Dominique Marie, Théophile Grébert, Christiane Bouchier, Sophie Le Panse, Martin Gachenot, Francisco Rodríguez, José L. Garrido

**Affiliations:** 1 Sorbonne Université, Centre National de la Recherche Scientifique, Station Biologique de Roscoff, UMR 7144, 29680 Roscoff, France; 2 Centre National de la Recherche Scientifique, Station Biologique de Roscoff, FR 2424, 29680 Roscoff, France; 3 CEA/Genoscope/LABGeM, Université d’Evry, CNRS UMR 8030 & Université Paris-Saclay, 91057 Evry, France; 4 Institut Pasteur, Genomics Platform, Biomics, 75015 Paris, France; 5 Instituto Español de Oceanografía, Centro Oceanográfico de Vigo, 36390 Vigo, Spain; 6 Instituto de Investigaciones Marinas (CSIC), 36208 Vigo, Spain

## Abstract

All characterized members of the ubiquitous genus *Acaryochloris* share the unique property of containing large amounts of chlorophyll (Chl) *d*, a pigment exhibiting a red absorption maximum strongly shifted towards infrared compared to Chl *a*. Chl *d* is the major pigment in these organisms and is notably bound to antenna proteins structurally similar to those of *Prochloron*, *Prochlorothrix* and *Prochlorococcus*, the only three cyanobacteria known so far to contain mono- or divinyl-Chl *a* and *b* as major pigments and to lack phycobilisomes. Here, we describe RCC1774, a strain isolated from the foreshore near Roscoff (France). It is phylogenetically related to members of the *Acaryochloris* genus but completely lacks Chl *d*. Instead, it possesses monovinyl-Chl *a* and *b* at a *b/a* molar ratio of 0.16, similar to that in *Prochloron* and *Prochlorothrix*. It differs from the latter by the presence of phycocyanin and a vestigial allophycocyanin energetically coupled to photosystems. Genome sequencing confirmed the presence of phycobiliprotein and Chl *b* synthesis genes. Based on its phylogeny, ultrastructural characteristics and unique pigment suite, we describe RCC1774 as a novel species that we name *Acaryochloris thomasi*. Its very unusual pigment content compared to other *Acaryochloris* spp. is likely related to its specific lifestyle.

## Introduction

Cyanobacteria, the oldest and one of the most diversified groups of photosynthetic microorganisms on Earth, have colonized virtually all environments reached by solar light^1^. The vast majority of cyanobacteria harvest photons using large antenna complexes called phycobilisomes, which are constituted of various assemblages of phycobiliproteins^2^. Because of their large size and water solubility, phycobilisomes are extrinsic to photosynthetic membranes and sit on top of thylakoid-embedded photosystems I and II^3^. Certain atypical cyanobacteria lack phycobilisomes and instead possess antenna complexes that are incorporated into membranes, like plants and most algae, although the structure of these antennae is different between prokaryotic and eukaryotic oxyphototrophs^4,5^. These atypical cyanobacteria include the Chl *a/b*-containing genera *Prochloron* and *Prochlorothrix* as well as *Prochlorococcus*, a ubiquitous marine cyanobacterium most abundant in warm oligotrophic areas and which contains divinyl derivatives of both Chl *a* and *b*^6^. Although these three cyanobacteria are sometimes erroneously gathered into the division *Prochlorophyta*, order *Prochlorales* and/or family *Prochloraceae*^7^, they are not phylogenetically closely related to one another^8–10^ and it is preferable to call them ‘green oxyphotobacteria’ by homology with ‘green algae’, to highlight their shared (mono- or divinyl) Chl *b* content with these eukaryotic phototrophs^11,12^.

More recently, a fourth atypical cyanobacterium, *Acaryochloris marina*, was also found to lack phycobilisomes but surprisingly its main photosynthetic pigment was Chl *d*, Chl *a* being present only in minor amounts within the cells^13,14^. Consequently, Chl *d* not only acts as a light-harvesting pigment in *A*. *marina* cells, but also replaces Chl *a* in reaction centers^15,16^. The unusual absorption properties of Chl *d*, characterized by a displacement of its major Q_y_ band towards infrared, enables *Acaryochloris* spp. to exploit an extended region of light beyond the visible spectrum and thus to thrive in habitats enriched in near-infrared radiation. After the initial discovery of *A*. *marina* in coral reefs of Palau^14,17^, Chl *d*-containing *Acaryochloris*-like cyanobacteria have been observed in a variety of environments: as epiphytes on red algae in rocky seashores and temperate mangroves, associated with coralline algae, in endolithic biofilms, on the surface of sediments in temperate and polar areas, and even in hypersaline eutrophic lakes^18–25^. Interestingly, Chl *d* was reported in the cyanobacterium *Chlorogloeopsis fritschii* that also contains Chl *f*, a pigment even more red-shifted than Chl *d*^26^.

Besides Chl *d*, the *A*. *marina* type strain MBIC11017 was also found to possess substantial amounts of phycocyanin (PC) and allophycocyanin (APC)^13,17^. These are organized as phycobiliprotein aggregates localized in electron-dense inter-thylakoidal areas^27–29^ and are able to efficiently transfer photon energy to photosystem II^30^. All three green oxyphotobacteria only possess phycobiliprotein remnants, i.e. phycoerythrin in *Prochlorococcus* (either α and β or β subunits only^31–33^), α− and β−PC in *Prochloron*^34^ and β−APC in *Prochlorothrix* (as found by screening the genome of *P*. *hollandica* PCC 9006^35^). These phycobiliproteins are only present at low to undetectable amounts in the cells and even if phycoerythrin was shown to play a minor role in light harvesting in *Prochlorococcus marinus* SS120, it is possible that they may rather act in light sensing, although this function has not yet been formally demonstrated^33^.

Here we describe RCC1774, the type strain of a new species that is phylogenetically related to the *Acaryochloris* genus, but which possesses Chl *a* as the major photopigment as well as Chl *b*, zeaxanthin, β,ε-carotene and PC as main accessory pigments. This is the first time that this suite of pigments is reported for a member of the *Acaryochloris* genus and for cyanobacteria at large. This novel species, that we propose to call *Acaryochloris thomasi* sp. nov. in honour of its isolator, Jean-Claude Thomas, is therefore the fourth ‘green oxyphotobacterium’ ever described, but the only one which possesses substantial amounts of PC. This discovery should thus provide interesting novel insights into the evolution of pigment synthesis in cyanobacteria.

## Results

### Phylogeny

**Figure 1:**
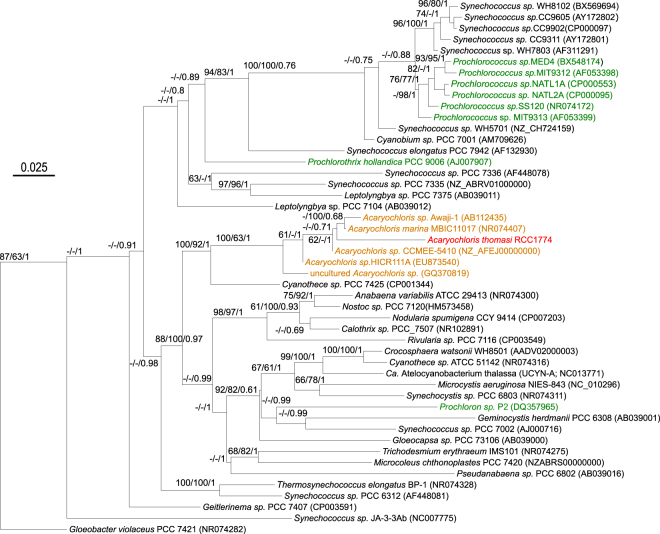
Maximum likelihood phylogenetic tree based on the 16S rRNA gene showing the position of the new RCC1774 strain compared to a diverse selection of freshwater and marine cyanobacteria. Green oxyphotobacteria are highlighted in green, while *Acaryochloris* spp. are shown in orange and the new strain in red. Numbers shown at nodes correspond to bootstrap values for ML, NJ analyses and Bayesian posterior probabilities (PP; ranging between 0 and 1), respectively. Only values higher than 60% for bootstrap analyses and 0.60 for PP are shown on the phylogenetic tree. For each strain, the sequence accession number of the 16S rRNA gene (or genome, if not available) is indicated between brackets. The scale bar represents 0.025 substitution per nucleotide.

The 16S rRNA gene sequence of strain RCC1774 was compared with sequences from a wide range of other cyanobacteria representative of the large genetic diversity existing within this ancient group (Fig. 1). All three tested phylogenetic methods (Maximum Likelihood/Neighbor-Joining/Bayesian inference) consistently placed RCC1774 within the *Acaryochloris* lineage with strong bootstrap support, even though its precise phylogenetic position within this clade was less well supported. The 16S rRNA gene sequence of RCC1774 sequence shares between 94.7 and 95.6% identity with the different members of the *Acaryochloris* genus (Supplementary Table S1) and is therefore more distant from all *Acaryochloris* strains available to date in culture than they are from one another (i.e. 97.5–99.3% nucleotide identity). Surprisingly, RCC1774 also shares 94.5% identity with strain PCC 7425, a distant and possibly incorrectly assigned member of the *Cyanothece* genus.

In order to validate the phylogenetic position of RCC1774, we also performed a phylogenomic analysis using a concatenation of 29 conserved core protein markers (Supplementary Fig. S1). The phylogenomic tree confirmed that RCC1774 is closely related to *Acaryochloris*, since it was found with a very high bootstrap support at the root of the only two other *Acaryochloris* strains available so far in the Genbank genome database. Such concatenated core markers offer a much better phylogenetic resolution than does the sole 16S rRNA gene, particularly for basal nodes, so that several poorly supported branches in the 16S rRNA gene tree fell at a different position in the phylogenomic tree. The topology of the latter tree was however fully consistent with previous phylogenomic analyses of the Cyanobacteria phylum^35–37^.

### Comparative HPLC pigment profiles

**Figure 2:**
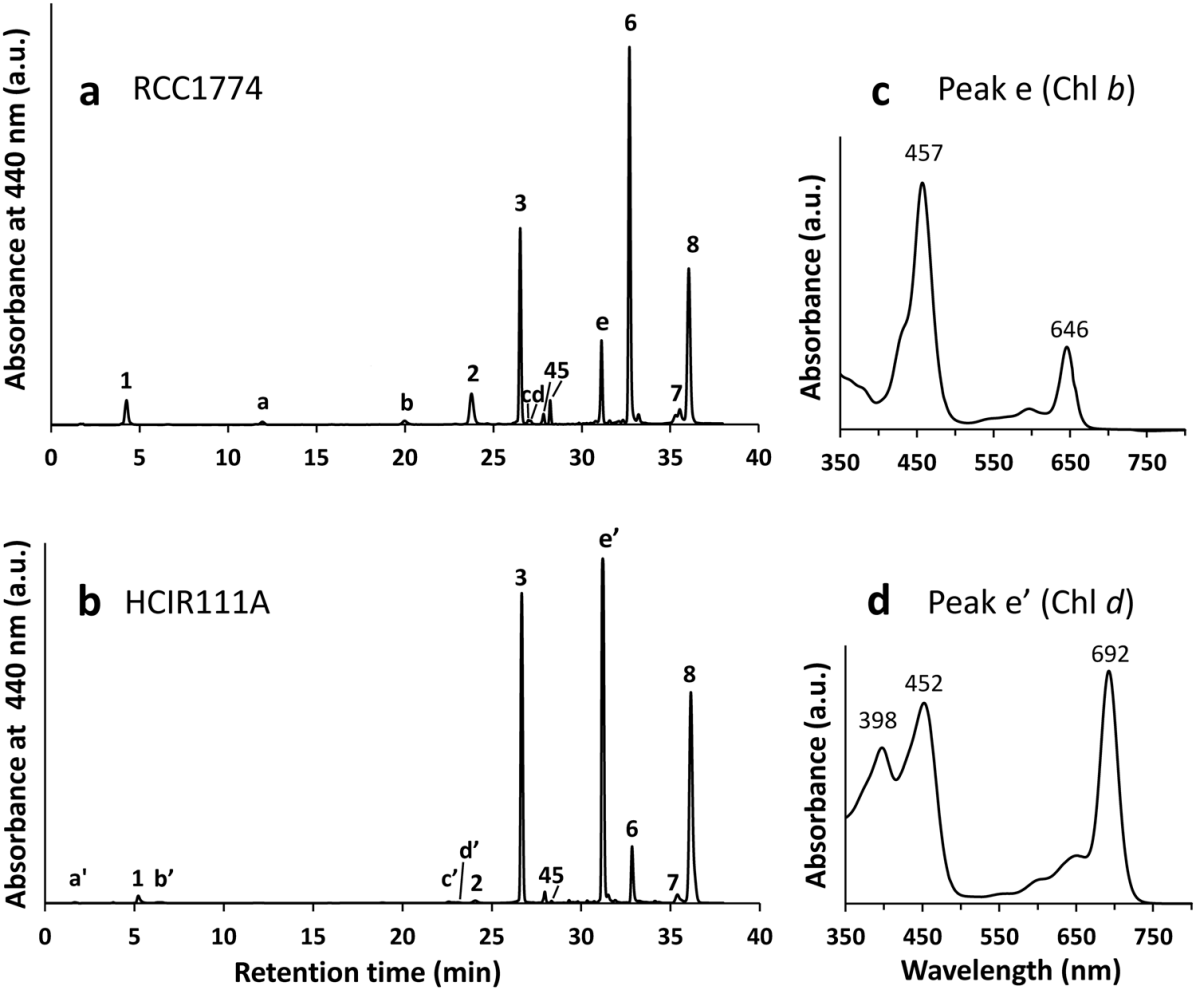
Comparative HPLC analysis of the pigment content of RCC1774 and the control Chl *d*-containing strain *Acaryochloris* sp. HCIR111A. (**a**,**b**) Chromatograms using the method described by Zapata and co-workers^38^. Pigments shared by the two strains (i.e. same retention time and absorption spectrum) are indicated by numbers: 1, Mg-2,4-divinyl pheoporphyrin a_5_; 2, possible caloxanthin (monohydroxy-zeaxanthin); 3, zeaxanthin; 4, 5 & 7, unidentified carotenoids; 6, Chl *a*; 8 β,ε-carotene (a.k.a. α-carotene). RCC1774-specific pigments: a, Chlide *a*; b, possible nostoxanthin; c & d, unidentified carotenoids; e, Chl *b*. HCIR111A-specific pigments: a′ & b′, Chlide *d*-like pigments; c′ & d′, unidentified carotenoids; e′, Chl *d*. (**c**,**d**) On-line absorption spectra of Chl *b* (RCC1774) and Chl *d* (HCIR111A); for other peaks, see Supplementary Fig. S2.

Pigment analyses performed independently at Vigo and Roscoff provided identical pigment composition for the two strains tested (RCC1774 and HICR111A; Fig. 2 and Supplementary Fig. S2). When analyzed with the method of Zapata and coworkers^38^, the chromatogram of RCC1774 (Fig. 2a) showed that it contains both Chl *a* and *b* at concentrations of ca. 26.7 and 4.2 fg cell^−1^, respectively (Chl *b*:*a* molar ratio ∼0.16). By comparison, HICR111A exhibited the Chl content expected for typical *Acaryochloris* spp.^13^, with Chl *d* as the main Chl (its exact concentration could not be quantified due to lack of a standard) and minor amounts of Chl *a* (0.6 fg per cell; Fig. 2b). Because Chl *d* and Chl *b* coeluted using this HPLC method, strains were also analyzed employing the method developed by Garrido and coworkers^39^, which allows a partial separation of these two pigments (Supplementary Fig. S3). Inspection of the on-line absorption spectra of the corresponding peaks for both strains allowed us to unambiguously conclude about the absence of Chl *d* in RCC1774 and of Chl *b* in HICR111A (Fig. 2c,d). Two small peaks with short retention times in the chromatogram of HICR111A (peaks a′ and b′ in Fig. 2b) exhibited a very similar spectrum to that of Chl *d* (Supplementary Fig. S2), suggesting that they correspond to a Chlide *d-*like compound, a Chl *d* by-product. Both RCC1174 and HCIR111A strains possessed low amounts of the Chl *c*-like pigment Mg-2,4-divinyl pheoporphyrin a_5_. They also shared the same two major carotenoids, zeaxanthin and β,ε-carotene (a.k.a. α-carotene), as well as a number of minor carotenoids, including a caloxanthin-like pigment (Fig. 2 and Supplementary Fig. S2). Yet, some minor carotenoids were seemingly specific of one or the other strain, including a putative nostoxanthin that was found only in RCC1774 (Fig. 2a,b and Supplementary Fig. S2).

### Spectrometric characterization of whole cells and extracts

**Figure 3:**
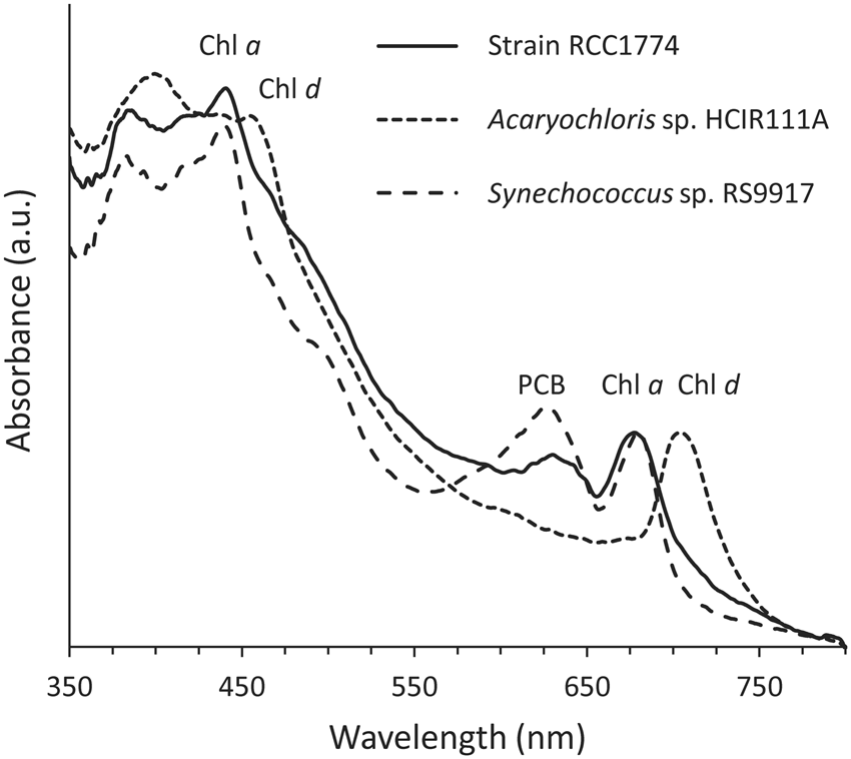
Whole cell absorption spectra of RCC1774 and the control strains *Acaryochloris* sp. HCIR111A and *Synechococcus* sp. RS9917. Prior to measurement, cells were disrupted using a French press to reduce light scattering.

As expected from their distinct Chl contents, absorption spectra obtained from broken cells of RCC1774 and HICR111A were totally different (Fig. 3). The latter strain exhibited the characteristic Chl *d* maxima at 455 and 705 nm, strongly red-shifted with respect to the corresponding peaks of Chl *a* (*A*_max_ at 440 and 677 nm, respectively) observed in both RCC1774 and the control *Synechococcus* sp. RS9917. Another striking difference between RCC1774 and HICR111A was a marked peak in the former but not the latter strain at a position similar to that of the phycocyanobilin peak of RS9917 (∼630 nm; Fig. 3). This strongly suggests the presence of substantial amounts of a PC-like phycobiliprotein in RCC1774 cells, although seemingly lower relative to Chl *a* than in cells of the typical blue-green cyanobacterium *Synechococcus* sp. RS9917, as judged from their different *A*_440 nm_ to *A*_630 nm_ ratios (Fig. 3). The presence of a PC-like pigment in RCC 1774 was confirmed by the occurrence of a maximum at ∼630 nm in the excitation spectra with emission set at 655 nm (C-PC emission maximum) or 680 nm (Chl *a* emission maximum; Supplementary Fig. S4A), similar to those observed for *Synechococcus* sp. RS9917, although the latter cells showed a much lower excitation maximum at 440 nm (Supplementary Fig. S4c). These data suggest that the PC-like pigment of RCC1774 can efficiency transfer energy to Chl *a*.

**Figure 4:**
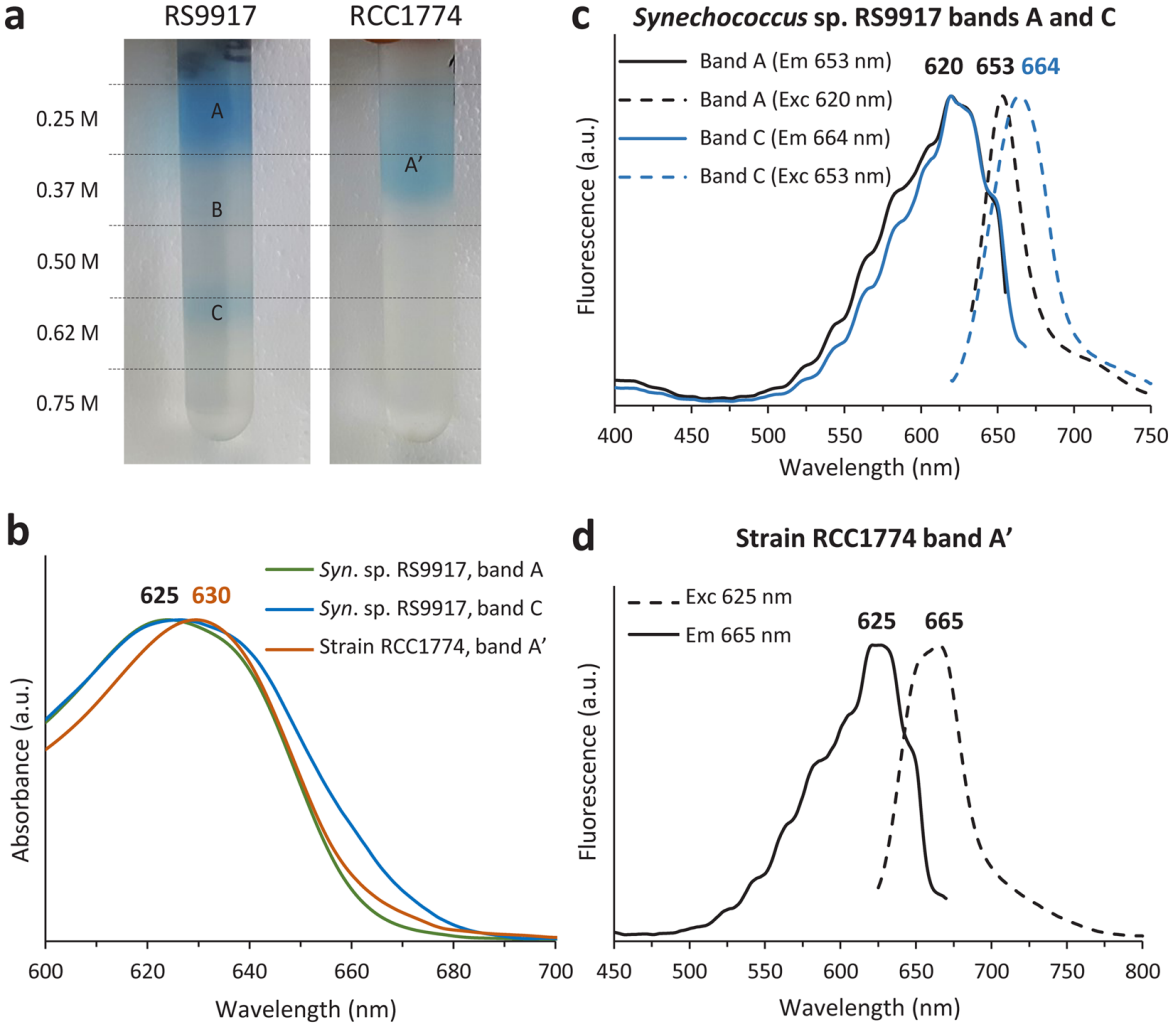
Characterization of water soluble extracts from cells of RCC1774 and the control strain *Synechococcus* sp. RS9917 separated by ultracentrifugation on a sucrose gradient. (**a**) Photograph of the blue-colored fractions obtained for RS9917 (left) and RCC1774 (right). (**b**) Absorption spectrum of the different fractions. (**c**,**d**) Excitation (plain lines) and emission spectra (dashed lines) of the different fractions obtained for RS9917 (**c**) and RCC1774 (**d**).

To further characterize the phycobiliprotein content of the novel strain, we made a water soluble extract of both RCC1774 and HCIR111A cells, followed by ultracentrifugation onto a sucrose gradient. A blue-coloured band (called A’) was obtained in the 0.37 M sucrose layer for RCC1774 (Fig. 4a), while no corresponding band was observed for HCIR111A (data not shown). By comparison, a partial dissociation of *Synechococcus* sp. RS9917 phycobilisomes produced two strong bands at 0.25 and 0.62 M, most likely corresponding to PC α − β monomers and entire phycobilisomes, respectively (bands A and C in Fig. 4a). A faint band (B) located between 0.37 and 0.50 M sucrose likely corresponded to phycobilisomes lacking C-PC hexamers. Thus, the pigment-protein complexes forming band A’ in RCC1774 appear to be slightly denser than C-PC monomers (band A) but much smaller than entire phycobilisomes (band C), suggesting that, like the type strain *A*. *marina* MBIC11017, RCC1774 does not contain complete phycobilisomes but only phycobiliprotein aggregates^28,29,40^. Both absorbance (Fig. 4b) and excitation spectra of these complexes showed a maximum that was red-shifted by 5 nm compared to the typical C-PC of *Synechococcus* sp. RS9917. Interestingly, the excitation spectrum of band A’ (Fig. 4d) also exhibited a marked shoulder at 650 nm and the position of the corresponding emission peak was located at 665 nm, like the whole phycobilisomes (band A) of *Synechococcus* sp. RS9917 and not at ∼653 nm like C-PC monomers (band C; Fig. 4c). This observation suggests that, besides PC, phycobiliprotein aggregates of RCC1774 also contain APC, albeit in a lesser amount than PC.

### Cell size and fluorescence characteristics

**Figure 5:**
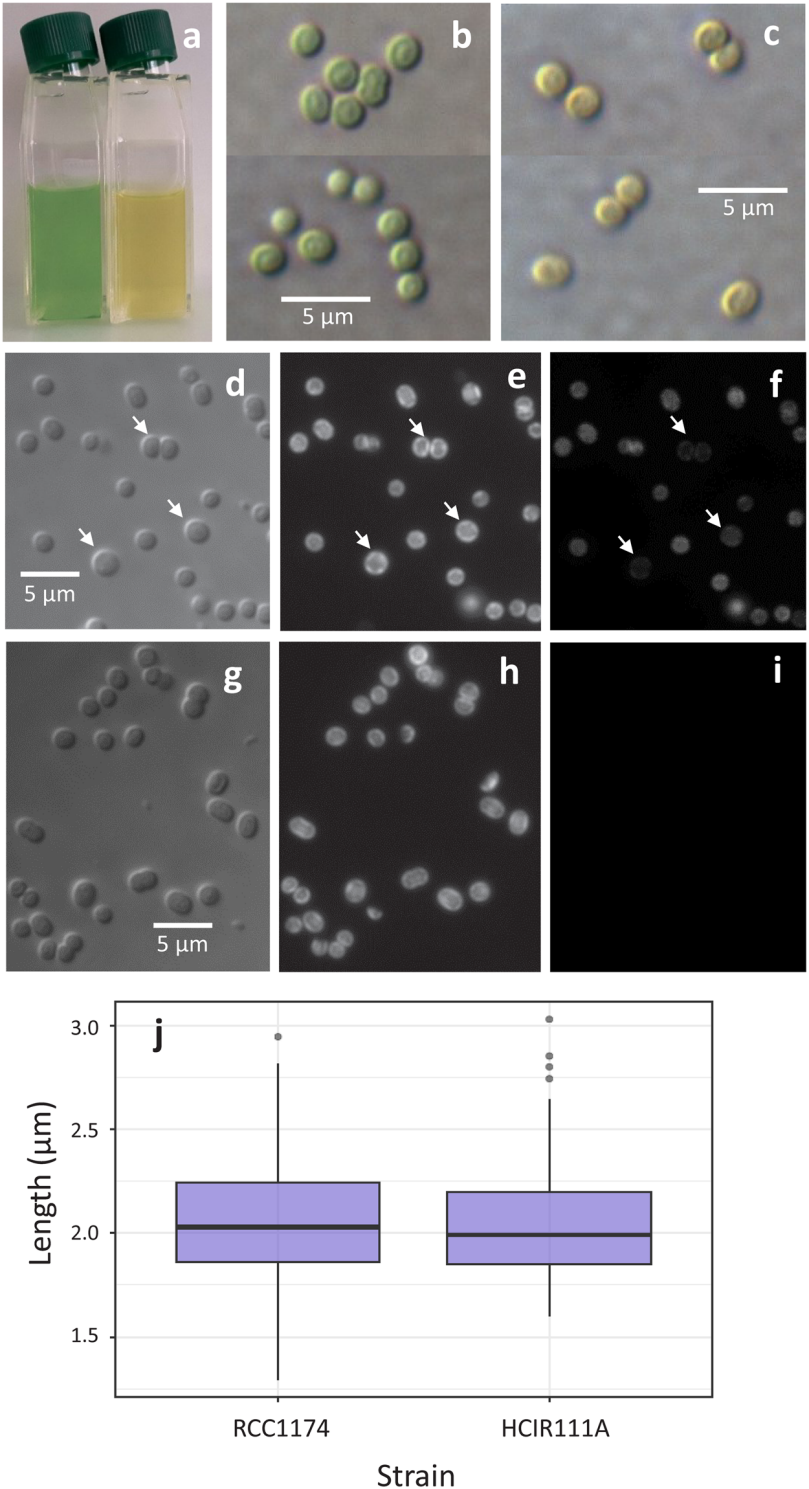
(**a**) Culture flasks of RCC1774 (left) and HCIR111A (right) showing their distinct colour. (**b**,**c**) Colour light micrographs of RCC1774 (**a**) and HCIR111A (**b**) using differential interference contrast (DIC). (**d**,**g**) Black and white light micrographs of RCC1774 (**d**) and HCIR111A (**g**) under DIC. (**e**,**f**) The same cells of RCC1774 **(e**,**f**) and HCIR111A (**h**,**i**) seen under epifluorescence using blue (**e**,**h**) and green (**f**,**i**) excitation. Arrows indicate three large or dividing cells that brightly fluoresce under blue (note the curtain-like organization of thylakoids) but comparatively less under green excitation. (**j**) Box plot chart showing the cell size distribution (n = 100) for each strain. The bold line indicates the median.

Cultures and individual cells of RCC1774 look green compared to the yellowish HCIR111A (Fig. 4a–c). RCC1774 cells are mostly coccoid but become ellipsoidal prior to cell division (Fig. 5b,d). Under the light microscope, cells have an average length of 2.05 ± 0.29 µm (s.d., n = 100), which is quite similar to *Acaryochloris* sp. HICR111A cells (Fig. 5j). It is worth noting that Mohr and coworkers^41^ reported a much smaller cell diameter for HCIR111A cells (0.74-1 µm), whereas the cell size reported here is in the upper range of that reported for other *Acaryochloris* spp. (1.5–2.0 µm cell diameter^14,24,42^). Under epifluorescence microscopy, both strains fluoresced red when excited by blue light (Fig. 5e,h), whereas only RCC1774 fluoresced red under green light excitation (Fig. 5f,i). This differential fluorescence might arise from the presence of Chl *b* and/or PC in RCC1774 but not HCIR111A cells, one or both of these pigments possibly being marginally excited by the light source due to the fairly large bandpass of the green filter of the epifluorescence microscope.

**Figure 6:**
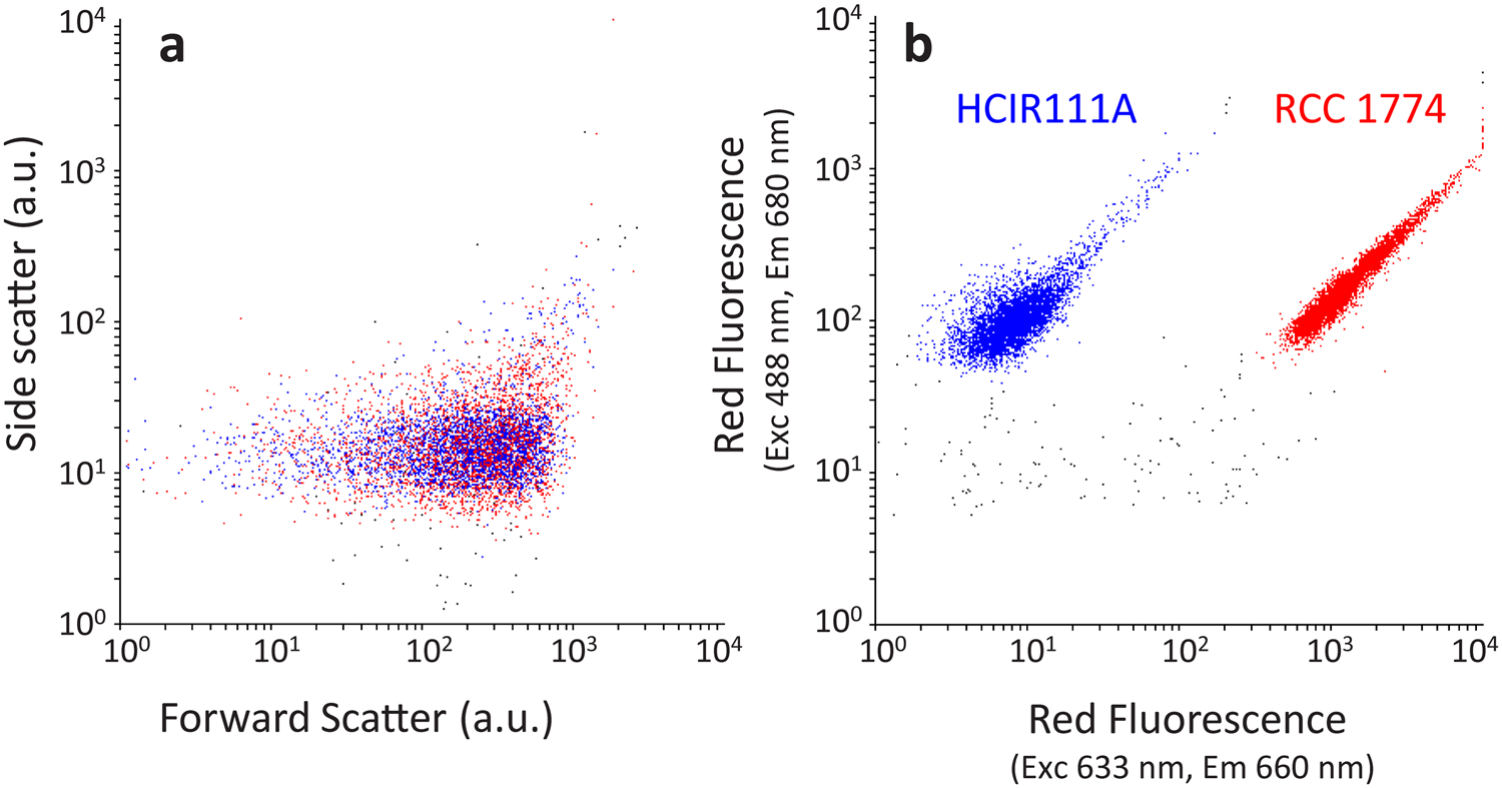
Flow cytograms of a mixture of RCC1774 and the control *Acaryochloris* sp. HCIR111A cultures. (**a**) Forward scatter vs. Side scatter cytogram. (**b**) Red fluorescence from the red laser (633 nm, exciting C-PC) vs. red fluorescence from the blue laser (488 nm, exciting Chls).

To complement these analyses, we also compared the flow cytometric signatures of RCC1774 and HCIR111A. These strains were indistinguishable on a forward scatter vs. side scatter cytogram (Fig. 6a), confirming that they have similar cell sizes. Cells of the two strains exhibited similar red fluorescence when excited using a blue (488 nm) laser (Fig. 6b *y*-axis) despite the different optical properties of their respective major Chls (see Supplementary Fig. S4 and Loughlin and coworkers^43^). In contrast, the flow cytometric signatures of RCC1774 and HCIR111A were completely different when excited using a red laser (633 nm; Fig. 6b *x*-axis), with RCC1774 exhibiting a red fluorescence signal (from its PC-like pigment) two orders of magnitude higher than HCIR111A.

### Cell ultrastructure

**Figure 7:**
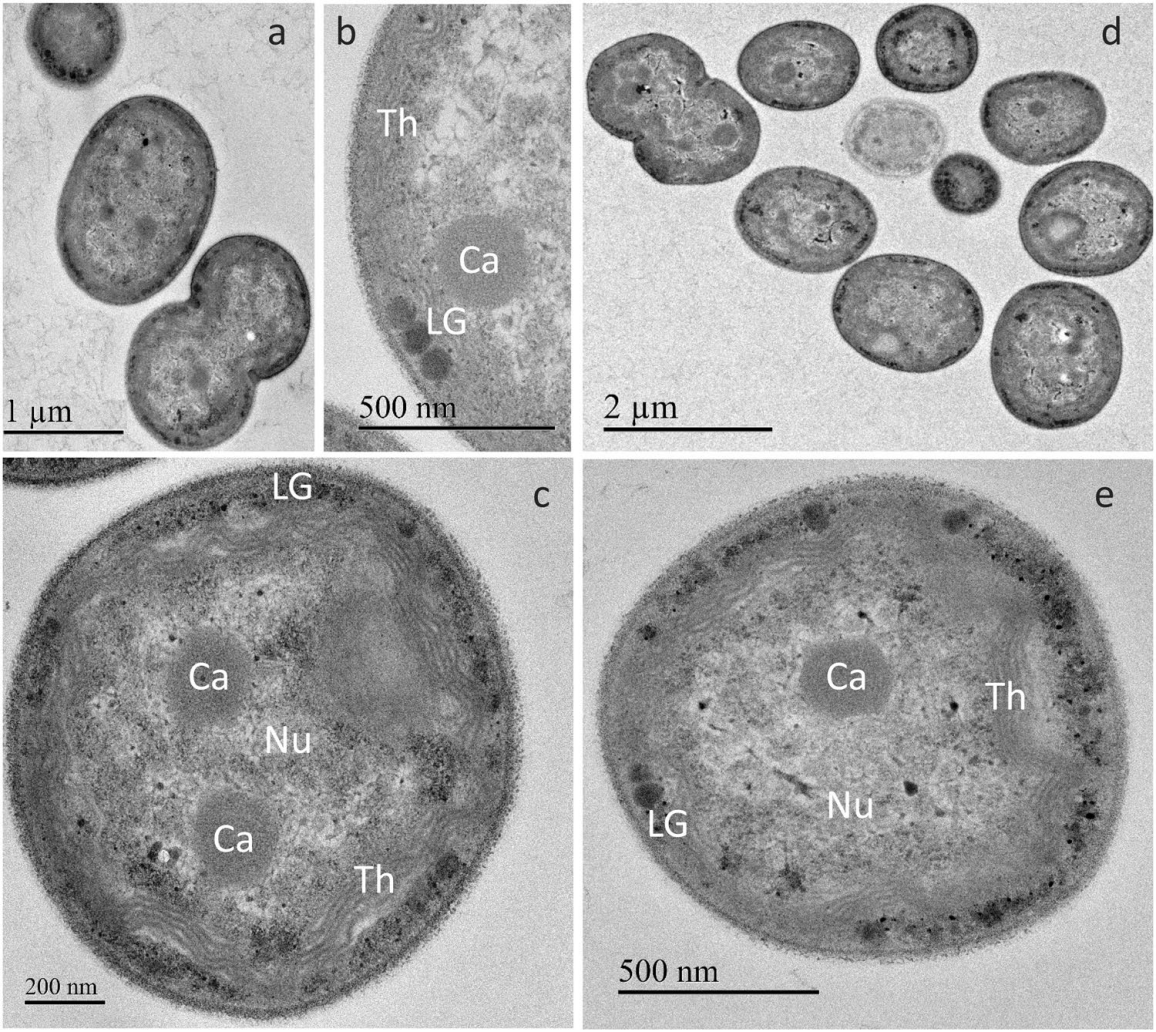
TEM images of RCC1774 (**a**–**c**) and the control *Acaryochloris* sp. strain HCIR111A (**d**,**e**). (**a**,**d**) Groups of cells; (**c**,**e**) Individual cells; (**b**) Detail of a RCC1774 cell. Abbreviations: Ca, carboxysomes, Nu, nucleoid; LG, lipid granules; Th, thylakoids.

In transmission electron microscopy (TEM), cells of RCC1174 were found to be covered with a fibrous outer membrane ∼15 nm thick (Fig. 7). TEM also revealed the occurrence of 4–5 undulated thylakoid layers located at the cell periphery, consistent with the curtain-like red fluorescent structures seen under epifluorescence microscopy (Fig. 5e). This suggests that thylakoids are attached to the cell membrane at regular points. However, we did not observe the channel-like structures that were shown to connect the central and peripheral cytoplasmic regions in the type strain *A*. *marina* MBIC11017^27–29^ nor observed any widened electron-dense inter-thylakoidal structures, suggested by Marquardt and coworkers^28^ to be the location of phycobiliprotein aggregates. Many small electron-dense particles were found between the thylakoids and the cell wall, probably corresponding to either lipid inclusions or glycogen granules, which sometimes merged into larger round granules of up to 80 nm diameter (Fig. 7b). The central part of the cell contains the nucleoid and 2 to 4 carboxysomes. This ultrastructure appears to be very similar to that of *Acaryochloris* sp. strain HICR111A (Fig. 7d–f), despite their different Chl content.

### Insights from genome analysis

In order to confirm findings about pigment synthesis from biochemical and spectroscopic analyses, a draft genome was obtained for RCC1774. The 72 contigs assigned to RCC1774 altogether totaled 5 931 831 bp with a G + C content of 50.2%. This compares well with *A*. *marina* MBIC11017 which has a chromosome size of 6 503 723 bp and G + C content of 47%^44^. Some of these contigs might correspond to plasmids (or plasmid fragments), since strain MBIC11017 was shown to contain nine plasmids, including one (pREB3) that contains most genes involved in the synthesis of C-PC^44^. Screening of the RCC1774 genome revealed the presence of one gene with strong homology to the Chlide *a* oxygenase (Cao) of *Prochloron* and *Prochlorothrix* (66 and 63% identity, respectively^45^). As expected, this gene is absent from all Chl *d*-containing *Acaryochloris* genomes sequenced thus far, the closest homolog in these genomes being pheophorbide *a* oxygenase (Pao) genes, which are also present in RCC1774 (Supplementary Table S2). Genomic searches also revealed the presence of seven Chl *a/b* light-harvesting protein genes (in addition to the photosystem II CP43 protein encoded by *psbC*) as well as a large set of phycobiliprotein genes (Supplementary Table S2). Besides the *cpcBA* operon encoding the two subunits of C-PC, the RCC1774 also contain *apcB* coding for the β-subunit of APC. A close homolog of genes annotated as ‘*apcA’* in *A*. *marina* MBIC11017^44^ and *Acaryochloris* sp. HCIR111A^41^ is also present in RCC1774 (C1752_02094). However, this gene and its homologs in other *Acaryochloris* spp. exhibit only low homology to *bona fide apcA* genes, encoding the α-subunit of APC (e.g. *slr2067* in *Synechocystis* sp. PCC 6803). Instead, they appear to be homologs of *Synechocystis slr0149*, which codes for a phycobiliprotein-related protein that, together with eight other genes from the same gene cluster (*slr0144-0152*), is thought to be involved in photosystem II assembly^46^. This functional assignment is further confirmed by the presence in the RCC1774 genome of homologs of *slr0144-0148* and *slr0151-0152* located in the immediate vicinity of *C1752_02094*, as is also the case for two out of the three genes annotated ‘*apcA’* in *A*. *marina* MBIC11017 (Supplementary Fig. S5), and it is therefore unlikely that this gene codes for an APC α-subunit. Additionally, RCC1774 contains six genes coding putative linker polypeptides, including four with similarity to the rod-core linker CpcG and two others coding for C-PC rod linkers CpcC and CpcD. At last, RCC1774 exhibits a complete set of phycocyanobilin lyases, including CpcE/F, CpcT and two CpcS homologs together with a putative CpcU, a protein that was shown to form a heterodimer with CpcS in *Synechococcus* sp. PCC 7002^47,48^.

## Discussion

Strain RCC1774 was characterized as a novel, distant member of the *Acaryochloris* genus that exhibits a unique pigment composition compared to all other isolates described so far in this genus^13,17,41,49^. Indeed, RCC1774 totally lacks Chl *d* and possesses both Chls *a* and *b* with a *b/a* ratio of ∼0.16, a value in the range of those reported previously in natural *Prochloron* spp. populations (0.08-0.39^50–53^) and *Prochlorothrix* spp. isolates (0.05–0.14^54,55^); RCC1774 is thus the fourth ‘green oxyphotobacterium’ described to date. These organisms belong to distinct genera that are widely dispersed across the cyanobacterial radiation (Fig. 1 and Supplementary Fig. S1), suggesting that the ability to synthesize Chl *b* has been acquired at least *five* times independently during the evolution of Cyanobacteria, if one also accounts the endosymbiotic ancestor of green algae that is often considered to be a Chl *b*-containing cyanobacterium^45^. It is worth mentioning in this context that Tsuchiya and coworkers^56^ attempted to transform *A*. *marina* MBIC11017 cells to make them artificially produce Chl *b* by introduction of the gene for Chlide *a* oxygenase. However, the transformant instead produced [7-formyl]-Chl *d*_P_, a form of Chl unknown in nature, which these authors hypothesized was produced by the combined action of Chlide *a* oxygenase and the product(s) of the yet-to-be characterized gene(s) involved in Chl *d* biosynthesis, which was/were not inactivated in the recombinant strain. This suggests that RCC1774 has evolved from a Chl *d*-producing *Acaryochloris*-like ancestor by acquiring a Chlide *a* oxygenase gene, while completely eliminating Chl *d* biosynthesis genes. Alternatively, the ancestor of all *Acaryochloris* spp. could have contained Chl *b* and RCC1774 might be a descendant of this ancient lineage, Chl *d* appearing only later during the evolution of this taxon. The topology of the phylogenomic tree (Supplementary Fig. S1) tends to support the second hypothesis, but this needs to be confirmed by including more members of the *Acaryochloris* lineage (as we did for the 16S rRNA tree; Fig. 1), when such genomes will be available. In any case, the genomic comparison of RCC1774 and Chl *d*-producing *Acaryochloris* spp. should help discover valid candidate(s) for Chl *d* synthase(s). In this context, although it has been suggested that *A*. *marina* MBIC11017 AM1_5665 might have this function^44^, the presence of a close homolog in RCC1774 (C1752_00555) somewhat invalidates this hypothesis, while two other proposed candidates (AM1_5023 and AM1_5798) have only distant homologs in RCC1774 and are therefore more likely to be involved in Chl *d* synthesis.

Another striking trait of RCC1774 is its high C-PC content. Amounts of the latter phycobiliprotein are highly variable among Chl *d*-containing *Acaryochloris* spp. studied so far^13,17,41,49^ and the content of RCC1774 appears to be in the upper range, like that of the *A*. *marina* type strain MBIC11017. Besides C-PC, the presence of an APC-like pigment in RCC1774 is also suggested here by characteristic shoulders in the RCC1774 fluorescence emission spectra of whole cells and water soluble extracts (Fig. 4d and Supplementary Fig. S4a). Analysis of the RCC1774 genome shows that it contains the whole set of genes necessary to synthesize C-PC but only a very partial set of APC genes, essentially consisting of one *apcB*-like gene, encoding an APC β-subunit, while there are seemingly no homologs of *apcC* through *apcF* (Supplementary Table S2), like in Chl *d*-containing *Acaryochloris* spp.^41,44^. We also retrieved a sequence in the RCC1774 genome that is homologous to a gene present in up to 4 copies in *Acaryochloris* spp. and that has been designated ‘*apcA’*^41,44^. However, its closer relatedness and shared genomic context with the photosystem II assembly gene *slr0149* of *Synechocystis* sp. PCC 6803 (Supplementary Fig. S5) suggest that this functional assignment might be erroneous. This raises the question of the structural arrangement of phycobiliprotein aggregates in RCC1774. While these are clearly comprising a typical C-PC, the role and organization of the vestigial APC remains obscure. In the absence of a core-membrane linker polypeptide (ApcE), the multi-copy CpcG-like linkers (Supplementary Table S2) probably have a critical role for the functioning of phycobiliprotein aggregates and notably for their connection to photosystems. *Anabaena* sp. PCC 7120 was reported to contain super-complexes consisting of one to three rod-shaped PC-CpcG3 aggregates specifically associating to tetrameric PSI complexes^57^. A similar organization is thus possible in RCC1774, given that three out of the four CpcG-like proteins of RCC1774 have, like *Anabaena* sp. CpcG3 (a.k.a. CpcL), a hydrophobic C-terminus^57^. The exact localization of these phycobiliprotein aggregates within RCC1774 cells however remains unclear, since we did not observe any electron-dense regions between thylakoid membranes, like those previously reported for *A*. *marina* MBIC11017^27^. Yet, the strong energetic coupling between these aggregates and photosystems indicates a localization in the immediate vicinity of the latter, i.e. either in the thylakoid stroma (like typical phycobilisomes) or possibly in the thylakoid lumen (like cryptophycean phycobiliproteins).

Most RCC1774 carotenoids were found to be shared with *Acaryochloris* sp. HCIR111A (Fig. 2). While zeaxanthin appears to be the dominant carotenoid in RCC1774 cells, as expected given the fairly high light conditions used here, the second most abundant carotenoid was β,ε-carotene (a.k.a. α-carotene), like in all *Prochlorococcus* and other *Acaryochloris* strains characterized to date^6,13^ and not β,β-carotene (a.k.a. β-carotene) like in *Prochloron*, *Prochlorothrix* and most typical cyanobacteria. This implies that RCC1174 must possess two carotene lycopene cyclase forms, β and ε, as previously demonstrated in *Prochlorococcus*^58^. RCC1774 indeed possesses a quite extensive set of carotenoid genes (Supplementary Table S2), including three lycopene cyclase homologs (CruA_1, CruA_2 and CruP) also found in *A*. *marina* MBIC11017^44^. Yet, compared to the latter strain, RCC1774 seemingly lacks a CrtL-b-type lycopene β−cyclase and possesses an additional putative zeaxanthin glucosyltransferase (CrtX; Supplementary Table S2).

The very unusual Chl content of RCC1774 compared to other *Acaryochloris* spp. was likely selected for by its specific lifestyle. All Chl *d*-containing *Acaryochloris* strains isolated so far were found to be associated with other organisms (mostly didemnid ascidians or red algae) and generally lived beneath these organisms, so that a large part of the incident solar light spectrum was filtered out, creating a light niche enriched in far-red wavelengths^43,59^. These conditions likely constitute a strong selective pressure favoring organisms containing Chl *d* and/or Chl *f*, two pigments well suited to harvesting the near-infrared end of the visible spectrum^26^. By comparison, RCC1774 was isolated from a benthic population living on a rocky substrate on the North coast of Brittany (France), and was apparently not associated with another organism. Surprisingly, its apparent benthic lifestyle in the field is somewhat contradicted by the fact that, in culture, RCC1774 cells live in suspension in the medium (even in the absence of stirring) and thus rather appear to be planktonic, in contrast to HCIR111A cells which tend to form aggregates surrounded by an extracellular matrix^41^ and often make a sticky layer at the bottom of culture flasks. The suite of pigments displayed by RCC1774 cells is consistent with a free-living lifestyle, as it can harvest a large part of the visible spectrum, notably blue and red-orange wavelengths (Fig. 3 and Supplementary Fig. S3).

In conclusion, the physiological and genetic characterization of strain RCC1774 led us to consider it as representing a new *Acaryochloris* species, further confirming the remarkable ubiquity of this atypical cyanobacterial genus as well as its wide genetic diversity and variety of lifestyles. Its formal diagnosis according to the botanical code follows, together with an emendation of the *Acaryochloris* genus.

## Taxonomic Description

### *Acaryochloris* Miyashita et Chihara emend. Partensky, Six, Ratin, Garczarek, Probert et Vaulot

#### Description

Cells are spheroidal or ellipsoidal. They are sheathed and non-motile. Most species contain Chl *d* as a major pigment and lack Chl *b*, but at least one species contains Chl *a* as a major pigment, possesses Chl *b*, and lacks Chl *d*. Phycobilisomes are absent. Thylakoids are appressed peripherally. Gas vacuoles are absent. Reproduction is performed asexually by binary division.

#### Type species

*Acaryochloris marina* Miyashita et Chihara in Miyashita *et al*.^14^ (authentic strain MBIC11017).

#### Etymology

A.cary.o.chlo’.ris. Gr. pref. *a*, without; Gr. comp. *caryo*-, nucleus-; Gr. adj. *chloros*, green; M.L. fem. n. *Acaryochloris*: without nucleus green.

### ***Acaryochloris thomasi*Partensky**, **Six**, **Ratin**, **Garczarek**, **Probert et D**. **Vaulot sp**. **nov**

#### Diagnosis

Cells non-motile and mostly solitary although they sometimes form clumps. Cells spheroidal, 1.4–2.4 µm in diameter, becoming elongated before division. Asexual reproduction by binary fission. Cells covered with a fibrous sheath. Thylakoid membranes undulated and located at the periphery of cells. Phycobilisomes absent. Cells contain chlorophylls *a* and *b*, zeaxanthin, α-carotene as well as phycocyanin as major pigments. Genome sequence (Genbank accession number: PQWO00000000).

#### Holotype

Figure 7c in this article.

#### Isotype

Cryopreserved culture strain RCC1774 in the Roscoff Culture Collection, Marine Biological Station, Roscoff, France.

#### Type locality

Atlantic Ocean, English channel, North of the *île verte*, situated in front of the Marine Biological Station of Roscoff, France (latitude: 48.732°N; longitude: 3.987°W).

#### Etymology

tho.ma.si. L. adj.: dedicated to Jean-Claude Thomas who isolated the type strain RCC1774.

## Materials and Methods

### Strains and culture conditions

All strains analyzed in the present study were obtained from the Roscoff Culture Collection^60^ (http://www.roscoff-culture-collection.org/). The novel *Acaryochloris* strain described here, RCC1774, was collected at low tide in February 1975 North of the *île verte* (Roscoff, France). The sample was enriched with Erdschreiber medium^61^ before cloning on plates with the same medium complemented with 1.5% agar. This strain was compared with the previously described HICR111A (RCC1983), a typical Chl *d*-containing *Acaryochloris* strain isolated from Heron Islands (Great Barrier Reef, Australia)^41^ and the marine *Synechococcus* sp. strain RS9917 (RCC556), a typical phycobilisome-containing, blue-green cyanobacterium, used as controls. Strains were grown in PCR-S11 medium^62^ at ca. 20.5 °C under an irradiance of 60–100 µmol photons m^−2^ s^−1^.

### High Performance Liquid Chromatography

The pigment composition of the abovementioned strains was analyzed both in the Roscoff and Vigo laboratories. In Roscoff, a 100 mL volume of culture of each strain was spun down and the pellet was resuspended in cold, pure methanol for 10 min. After two successive centrifugations at 20 000 × *g* in order to get rid of all debris, the supernatants were brought to 10% Milli-Q water and injected in a Hewlett Packard 1100 HPLC chain. All manipulations were done at 4 °C under dim light. The separation of the pigments was carried out following a modified version of the method of Zapata and coworkers^38^, using a pyridine solution at 0.05 M. In Vigo, approximately 10 mL of late exponential or early stationary phase culture were filtered in low light onto a glass fiber GF/F filter (Whatman, Maidstone, UK) without vacuum. Filters were protected from light at all processing stages and immediately frozen and stored at −20 °C. Frozen filters were extracted under low light in polytetrafluoroethylene (PTFE)-lined screw capped tubes with 5 ml 90% acetone using a stainless steel spatula for filter grinding. Tubes were chilled in a beaker of ice and sonicated for 5 min in an ultrasonic bath filled with an ice-water mix. Extracts were then filtered through 25 mm diameter syringe filters (MFS HP020, 25 mm, and 0.2 μm pore size, hydrophilic PTFE) to remove cell and filter debris. Before injection, 1 mL of each sample extract was supplemented with 0.4 ml of Milli-Q water to avoid peak distortion. Pigment extracts were analyzed by two previously described HPLC methods^38,39^. Pigments were separated using a Waters Alliance HPLC System (Waters Corporation) consisting of a 2695 separation module and a Waters 996 diode-array detector (1.2 nm optical resolution). Pigments were identified either by co-chromatography with authentic standards obtained from SCOR reference cultures^63^ or by diode-array spectroscopy. After checking for peak purity, spectral information was compared with a library of Chl and carotenoid spectra^64^. The Chl *d* spectrum was compared with that of reference data^13^.

### Spectrometric and flow cytometric measurements

Whole cell absorption spectra were recorded on a *mc²* spectrophotometer (SAFAS, Monaco) after cell disruption using a French press system, followed by centrifugation at 5000 × *g* to remove non-broken cells. *In vivo* excitation and emission fluorescence spectra were recorded with a LS-50B spectrofluorometer (Perkin Elmer, USA).

Flow cytometry measurements were made using a FACS Canto II Flow cytometer (Becton Dickinson, San Jose, CA, USA) equipped with two lasers emitting at 488 nm and 633 nm. The red emission fluorescence from the 488 nm laser was collected through a 610 Long Pass filter while that of the 633 nm laser through a 660/20 Band Pass filter.

### Phycobiliprotein analyses

2-L cultures were harvested in late exponential phase and centrifuged at 9 000 × *g*. In order to obtain typical phycobilisomes in a partially dissociated state, cells of *Synechococcus* sp. RS9917 were washed and resuspended in 0.55 M phosphate buffer. RCC1774 and *Acaryochloris* sp. HCIR111A cells were submitted to the classical procedure in 0.75 M phosphate buffer, as previously described^65^. All buffers contained a cocktail of antiproteases. Cells were broken using a cell disrupter at 1.6 kBar (Constant Systems Ltd.) and membranes were solubilized at room temperature in the presence of 5% Triton X-100 (Sigma Aldrich). After phase separation, the intermediate layer was loaded on a discontinuous sucrose gradient and ultracentrifuged at 40 000 × *g* overnight at 12 °C. The different colored bands were characterized by their absorption and fluorescence spectra as described above.

### Light and electron microscopy

Cells of both strains were imaged using an Olympus BX51 epifluorescence light microscope using an X100 objective, a SPOT RT camera and a fluorescent light source. Size measurements were made manually on one hundred cells from each strain using the Image J software (https://imagej.nih.gov/ij/) and plotted with R (https://www.r-project.org/).

For TEM preparations, 50 ml of dense cultures of both RCC1774 and *Acaryochloris* sp. HCIR111A strains were centrifuged for 3 min at 700 × *g* then fixed for 2 h in 0.2 M sodium cacocylate buffer (pH 7.2) with 2.5% glutaraldehyde. Cells were then washed three times in cacodylate buffer containing decreasing concentrations of sucrose (0.25 M, 0.12 M, 0 M) for 15 min each time, followed by postfixation for 1 h at 4 °C in 2% osmium tetroxide in 0.1 M cacodylate buffer. After three rinses in 0.2 M cacodylate buffer, cells were embedded in 2% agar, dehydrated by successive transfers through a graded ethanol series (25%, 50%, 70%, 90%, 3 × 100%), then embedded in Spurr’s resin. Sections were cut using a diamond knife on a Leica Ultracut UCT ultramicrotome (Leica, Wetzlar, Germany) and after staining with 2% uranyl acetate for 10 min and 2% lead citrate for 3 min, grids were examined with a Jeol 1400 transmission electron microscope (Jeol, Tokyo, Japan).

### DNA extraction and genome sequencing

Cells of a 2-L culture in exponential phase were centrifuged at 7 700 × *g* for 15 min and stored at −80 °C. After thawing, cells were resuspended in Tris-EDTA buffer pH 8.0 (10 mM Tris, 1 mM EDTA) and lysed in lysozyme 1 mg.mL^−1^ for 30 min at 37 °C and 45 × *g*. SDS (20% w/v) and proteinase K (20 mg.mL^−1^) were added to a final concentration of 0.5% and 200 μg.mL^−1^, respectively. After 5 h incubation at 55 °C and 45 × *g*, another proteinase K treatment at half of the initial concentration was conducted for 2.5 h. Proteins were removed by two extractions with phenol:chloroform:isoamyl alcohol (25:24:1), followed by a chloroform:isoamyl alcohol (24:1) treatment, using phase lock gel (QuantaBio). RNA was then removed by adding RNase A (Sigma Aldrich) at a final concentration of 100 µg.mL^−1^ at 37 °C for 30 min, followed by another phenol:chloroform:isoamylic alcohol (25:24:1) and chloroform:isoamylic alcohol (24:1) treatment. DNA was precipitated with 0.6 volume of cold isopropanol. The pellet was then successively washed with 100% ethanol and 70% and DNA was resuspended in Tris-EDTA, loaded onto an Amicon Ultra-4 column (100 000 MWCO; Millipore UFC810024) before elution in Tris-EDTA buffer.

The whole-genome sequencing was performed by the Genomics Platform of the Pasteur Institute. Briefly, the high throughput sequencing library was made using the NEXTflex PCR-Free DNA-Seq kit (Bioo Scientific, Austin, TX, USA), then was sequenced on a MiSeq System (Illumina, San Diego, CA, USA), which generated 160 bp paired-end reads, providing a mean coverage of 20X. *De novo* assembly and automatic functional annotation were subsequently done using the Sequana suite of NGS pipelines^66^, except for genes reported in Supplementary Table S2 that were manually annotated. After filtering out contigs smaller than 1 000 bp and retaining only contigs that hit cyanobacteria according to Blast analyses against the Genbank database, the RCC1774 draft genome encompassed 72 contigs ranging in size from 1 031 bp to 605 849 bp and was deposited in Genbank under accession number PQWO00000000.

### 16S rRNA phylogeny

A multiple alignment of 49 sequences of 16S rRNA genes from a selection of marine and freshwater cyanobacteria was generated with MAFFT v7.017^67^ followed by manual refinement using the Geneious software (Biomatters Ltd, Auckland, New Zealand). Phylogenetic reconstructions were performed using three different methods: Maximum Likelihood (ML), Neighbour Joining (NJ) and Bayesian inference. Jmodeltest 2.1.4 determined GTR + I + G as the best model according to the Akaike Information Criterion^68^. ML inference was done using PhyML v3.1^69^ with invariant sites and gamma distribution, NJ analyses using Phylip 3.69^70^ and Bayesian inference using MrBayes 3.1.2^71^. Four Markov Chain Monte Carlo simulations were run for 1.5 million generations that were sampled every 100 generations, the first 3 750 trees being discarded. The topology of the tree was obtained from ML analyses and the robustness of inferred topologies was supported by 100 non-parametric bootstrap samplings for ML and NJ and Bayesian posterior probabilities.

### Phylogenomic analysis

A maximum likelihood tree was generated by PhyML 3.1^69^ using an alignment of 29 concatenated core proteins (DnaG, Frr, NusA, Pgk, PyrG, RplA, RplB, RplC, RplD, RplE, RplF, RplK, RplL, RplM, RplN, RplP, RplS, RplT, RpmA, RpoB, RpsB, RpsC, RpsE, RpsI, RpsJ, RpsK, RpsM, RpsS and SmpB) after removal of ambiguous and saturated positions from each protein alignment, as previously described^36^.

### Data availability

All data in this article will be provided upon request.

## Electronic supplementary material

Supplementary Figures

Supplementary Table S1

Supplementary Table S2
