# Supplementary material for: A novel species of the marine cyanobacterium Acaryochloris with a unique pigment content and lifestyle
Source: Sci Rep. 2018 Jun 14;8:9142. doi: 10.1038/s41598-018-27542-7 (PMC6002478; doi:10.1038/s41598-018-27542-7)
Supplement: Supplementary file 1 — Supplementary Figures [file 41598_2018_27542_MOESM1_ESM.pdf]

Supplementary information : Supplementary Figures S1 to S5

(Supplementary Tables S1 and S2 are provided as separate .xlsx spreadsheets)

A novel species of the marine cyanobacterium *Acaryochloris* with a unique pigment content and lifestyle

Frédéric Partensky[1*], Christophe Six[1], Morgane Ratin[1], Laurence Garczarek[1], Daniel Vaulot[1], Ian Probert[2], Alexandra Calteau[3], Priscillia Gourvil[2], Dominique Marie[1], Théophile Grébert[1], Christiane Bouchier[4], Sophie Le Panse[2], Martin Gachenot[2], Francisco Rodríguez[5] & José L. Garrido[6]

[1]Sorbonne Université, Centre National de la Recherche Scientifique, Station Biologique de Roscoff, UMR 7144, 29680 Roscoff, France.

[2]Centre National de la Recherche Scientifique, Station Biologique de Roscoff, FR 2424, 29680 Roscoff, France.

[3]CEA/Genoscope/LABGeM, Université d'Evry, CNRS UMR 8030 & Université Paris-Saclay, 91057 Evry, France

[4]Institut Pasteur, Genomics Platform, Biomics, Paris, France.

[5]Instituto Español de Oceanografía, Centro Oceanográfico de Vigo, 36390 Vigo, Spain.

[6]Instituto de Investigaciones Marinas (CSIC), 36208 Vigo, Spain.

*Correspondence should be addressed to F.P. (email: frederic.partensky@sb-roscoff.fr)

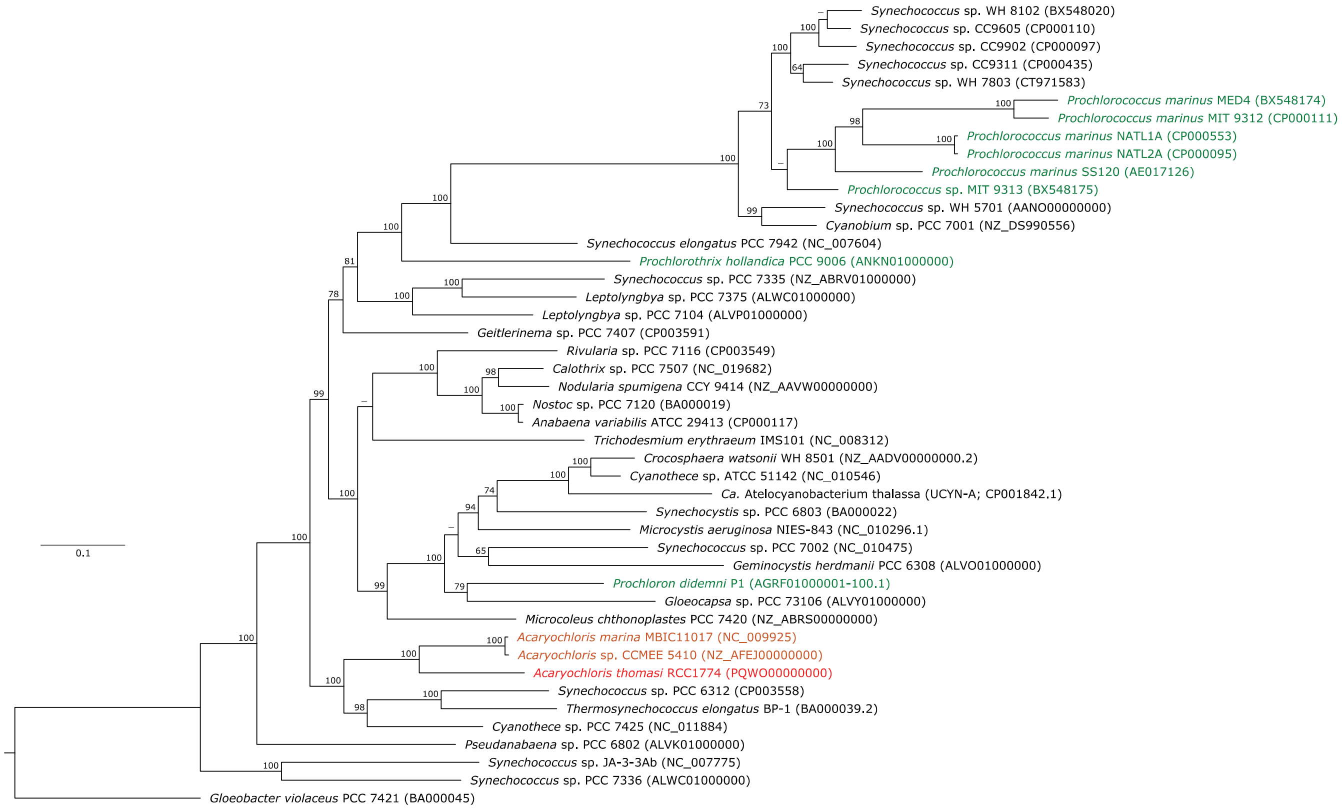

Supplementary Figure S1. Phylogenomic analysis of strain RCC1774. A maximum likelihood tree was generated using an alignment of 29 concatenated core proteins obtained from the same strains as for the 16S rRNA tree (Fig. 1) except those (mainly *Acaryochloris* spp.) for which the genome was either missing or of insufficient quality. Only bootstrap values higher than 60% are indicated. Green oxyphotobacteria are highlighted in green, while *Acaryochloris* spp. are shown in orange and the new strain in red. Genbank genome accession numbers are indicated between brackets after strain names. The scale bar represents 0.1 substitution per amino acid position.

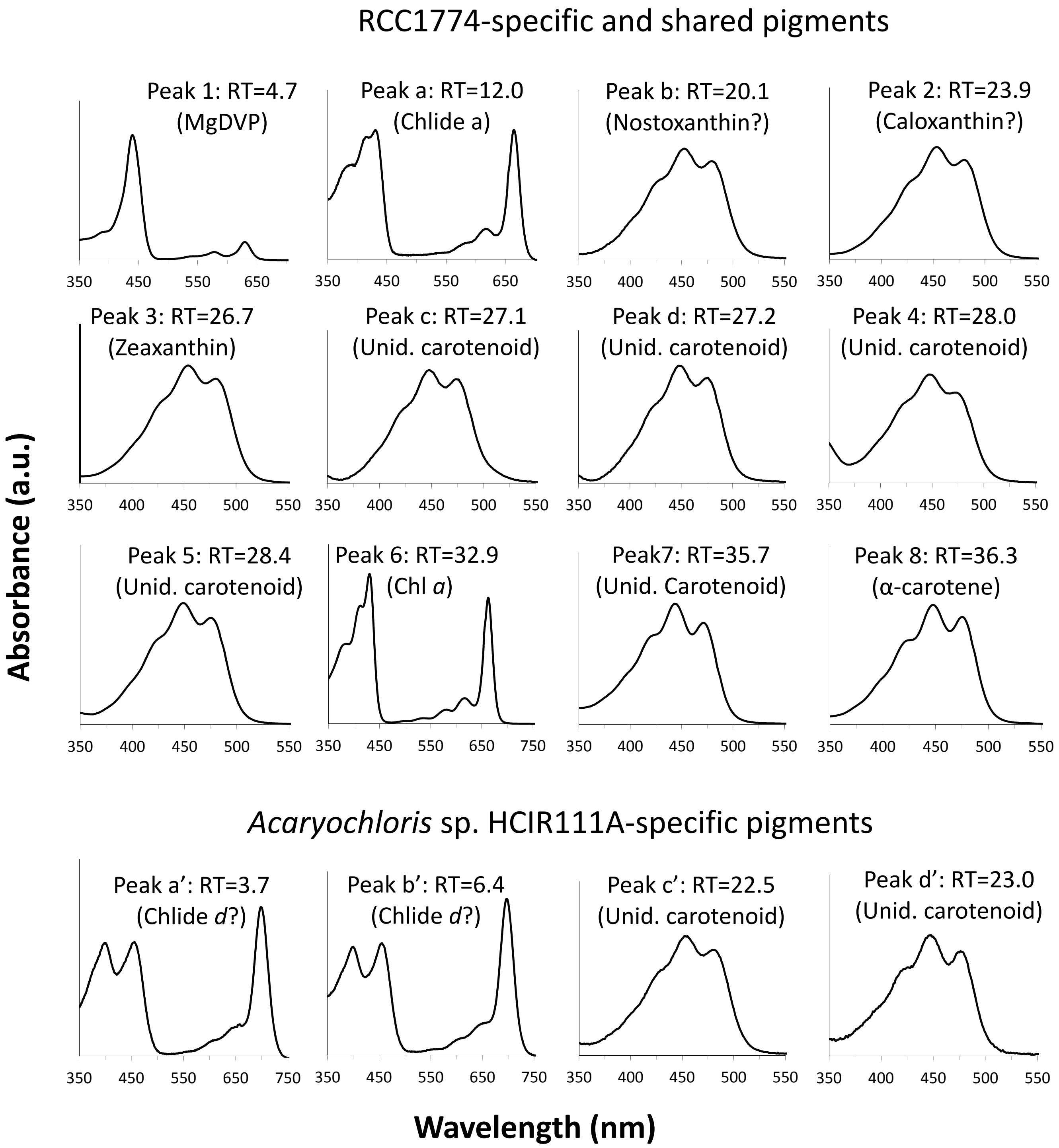

Supplementary Figure S2. On-line absorption spectra for all peaks of the HPLC chromatograms of strain RCC1774 and the control *Acaryochloris* sp. HCIR111A (except those already shown on Fig. 2) sorted by retention times. Spectra for pigments shared by the two strains are shown only once. Abbreviations: MgDVP, Mg-2,4-divinyl pheoporphyrin; RT, retention time (in min); Unid., Unidentified.

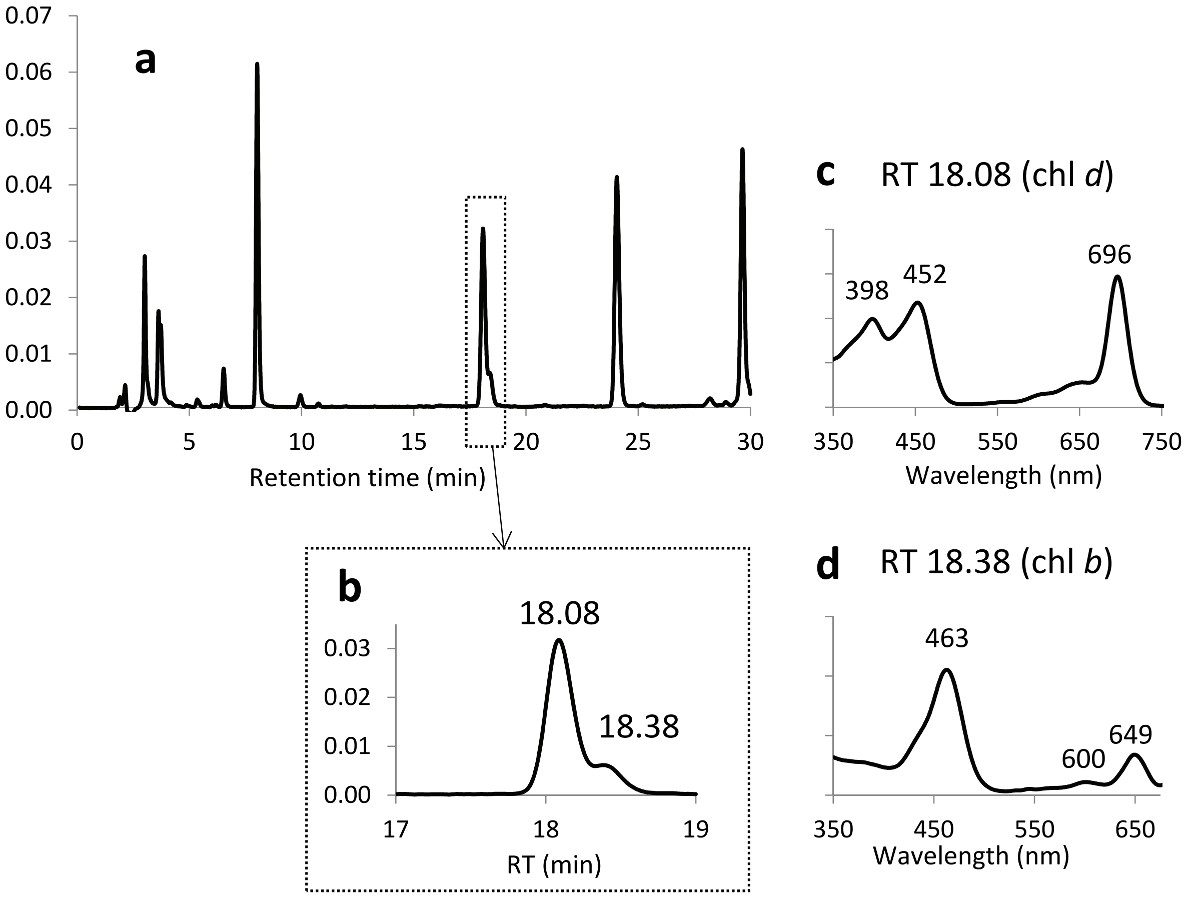

Supplementary Figure S3. HPLC chromatogram of a mixture of acetone extracts from strains RCC1774 and HCIR111A analyzed by the method developed by Garrido et al. [41] and on-line absorption spectra for the overlapping Chl *b* and *d* peaks. (a) Complete chromatogram. (b) Detail of the overlapping Chl *b* and *d* peaks and (c-d) corresponding on-line absorption spectra. RT, retention time.

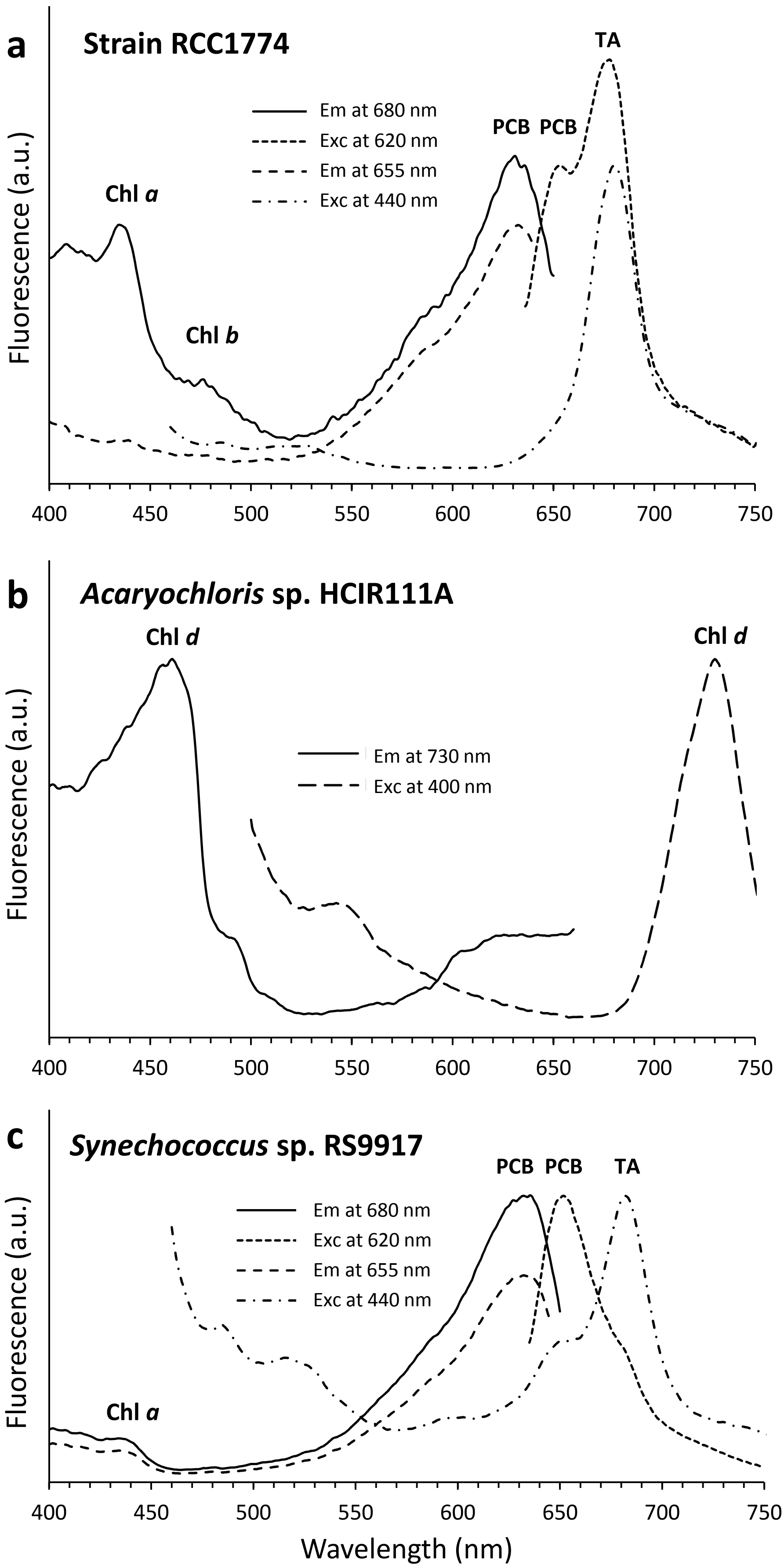

Supplementary Figure S4. Fluorescence spectra of whole cells of strain RCC1774 and the control strains *Acaryochloris* sp. HCIR111A and *Synechococcus* sp. RS9917. (a) RCC1774: Excitation (Exc) spectra with emission (Em) at 620 and 655 nm and Em spectra with Exc at 440 and 620 nm. (b) HCIR111A: Exc spectrum with Em at 730 nm and Em spectrum with Exc at 400 nm. (c) RS9917: Exc spectra with Em at 655 and 680 nm and Em spectra with Exc at 440 and 620 nm. PCB, phycocyanobilin, TA, terminal acceptor (Chl *a* and allophycocyanin).

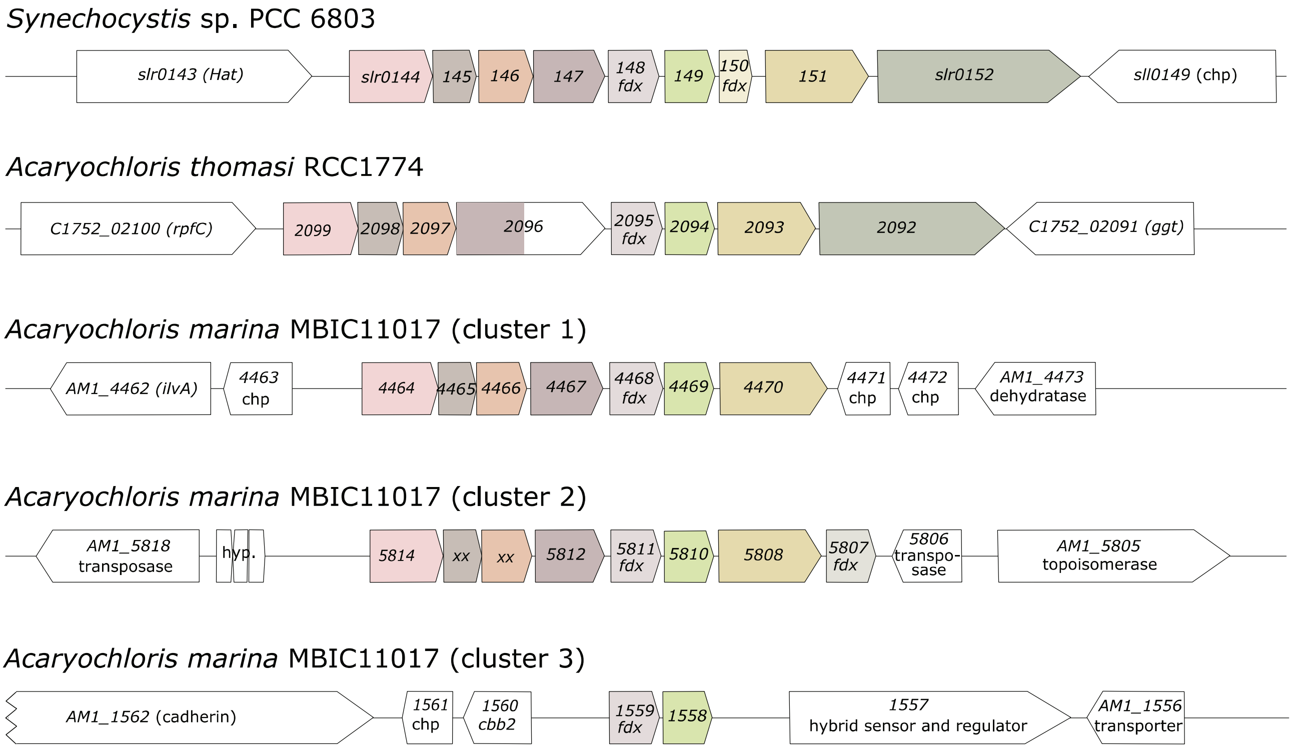

Supplementary Figure S5. Genomic context of the genes of *Acaryochloris marina* MBIC11017 called '*apcA*' by Swingley and coworkers[44] (*AM1_4469, AM1_5810 and AM1_1558*) and their homologs in *Synechocystis* sp. PCC 6803 (*slr0149*) and RCC1774 (*C1752_2094*). In *Synechocystis*, the gene cluster extending from *slr0144* to *slr0152* was suggested to be involved in photosystem II assembly[46]. Strain RCC1744 contains an almost complete gene cluster, including a homolog of the kinase gene *slr1052*, but it lacks a *slr0150* homolog, encoding a 2Fe-2S ferredoxin-type iron-sulfur binding domain protein, like *slr0158*. Note that the homolog of *slr0147*, which codes for a protein containing a 4-vinyl reductase domain (like *slr0144*), has an unusual 3'-end extension in RCC1774. *A. marina* MBIC11017 contains two such gene clusters, both lacking homologs of *slr0150* and of the kinase; yet the second one (*AM1_5814-5807)*, which is located between two transposases, contains two homologs of *slr0148 (AM1_5911 and 5807)*. This strain also contains a third cluster, in which only the *slr1058* and *slr0159* homologs are conserved. The color of a gene indicates its homology to the corresponding gene in *Synechocystis*, whereas a white background indicates that the gene has no (syntenic) homolog in *Synechocystis*. Numbers correspond to locus tags in the different strains. The two genes of *A. marina* MBIC11017 annotated 'xx' are present in the genome but were not initially modelled, whereas *AM1_5809*, a probable wrong prediction, is not shown. *chp*, gene coding for a conserved hypothetical protein; *hyp.*, putative gene (hypothetical); other abbreviations correspond to gene names.
